# Cross-Serological Reaction of Glandless Cottonseed Proteins to Peanut and Tree Nut Allergic IgE

**DOI:** 10.3390/molecules28041587

**Published:** 2023-02-07

**Authors:** Christopher P. Mattison, Zhongqi He, Dunhua Zhang, Rebecca Dupre, Steven W. Lloyd

**Affiliations:** 1 USDA-ARS, Southern Regional Research Center, New Orleans, LA 70124, USA; 2 USDA-ARS, Aquatic Animal Health Research Unit, Auburn, AL 36832, USA; 3 Oak Ridge Institute for Science and Education, U.S. Department of Energy, Oak Ridge, TN 37831, USA

**Keywords:** glandless, cottonseed, cross-reaction, vicilin, legumin, immunoglobulin E (IgE), peanut, tree nut, food allergy

## Abstract

Food allergy is a potentially life-threatening health concern caused by immunoglobulin E (IgE) antibodies that mistakenly recognize normally harmless food proteins as threats. Peanuts and tree nuts contain several seed storage proteins that commonly act as allergens. Glandless cottonseed, lacking the toxic compound gossypol, is a new food source. However, the seed storage proteins in cottonseed may act as allergens. To assess this risk, glandless cottonseed protein extracts were evaluated for IgE binding by peanut and tree nut allergic volunteers. ELISA demonstrated that 25% of 32 samples had significant binding to cottonseed extracts. Immunoblot analysis with pooled sera indicated that IgE recognized a pair of bands migrating at approximately 50 kDa. Excision of these bands and subsequent mass-spectrometric analysis demonstrated peptide matches to cotton C72 and GC72 vicilin and legumin A and B proteins. Further, in silico analysis indicated similarity of the cotton vicilin and legumin proteins to peanut vicilin (Ara h 1) and cashew nut legumin (Ana o 2) IgE-binding epitopes among others. The observations suggest both the cotton vicilin and legumin proteins were recognized by the nut allergic IgE, and they should be considered for future allergen risk assessments evaluating glandless cottonseed protein products.

## 1. Introduction

Gossypol glands, present in glanded (Gd) cottonseed (Gossypium), contain gossypol [1]. Gossypol is a phenolic compound that is toxic to humans, ruminants, and poultry and prevents the use of Gd cottonseed in food and animal feed. Identification of natural and induced forms of cottonseed with reduced levels of gossypol began in the 1960s, and continued research and breeding has led to the generation of glandless (Gl) cottonseed with much less gossypol content (e.g., 3.75 g/kg in Gd vs. 0.06 g/kg in Gl) [2]. Gl cottonseed has raised hopes that it may be used in an increased capacity for feed and food applications as a good source of fiber, oil, and protein. In 2019, the United States Food and Drug Administration approved the use of a Gl cottonseed strain (TAM66274) with ultra-low gossypol content in human and animal food applications [3]. Further, chemical and gravimetric analysis indicates that protein, starch, and phosphorus content is slightly higher in Gl cottonseed compared to Gd [2]. Mass-spectrometric analysis also indicates that there are differences in the content of some proteins, including legumin, 2 S albumin, and ‘vicilin-like antimicrobial peptides’, in Gl cottonseed compared to Gd [4,5]. However, another important consideration prior to using Gl cottonseed in large scale human food applications is the potential risk of food allergy.

Food allergy is a potentially life-threatening medical condition mediated by immunoglobulin E antibodies [6]. Food allergy can be very detrimental to family finances, emotional stability, and social standing [7,8]. The reported incidence of food allergy has increased over the past few decades [9,10]. Eight foods (milk, eggs, fish, shellfish, soybeans, wheat, peanuts, and tree nuts) that commonly cause food allergies have been recognized by the US Food and Drug Administration and they require labeling when included in foods [11]. However, there are reports of numerous foods that have the potential to cause food allergy [12]. For example, in the United States the Food Allergy Safety, Treatment, Education and Research (FASTER) Act will require, beginning January 2023, that sesame be included as the ninth major source of food allergens [13].

Peanuts and tree nuts contain several conserved seed storage proteins that commonly act as allergens, including legumin, vicilin, and albumin proteins [14,15,16]. Legumin and vicilin proteins belong to the cupin superfamily and contain the characteristic β-barrel structure. Legumin proteins are composed of acidic and basic subunits, held together by cysteine disulfide bonds, that assemble as hexamers into large molecular weight complexes [17]. Vicilins are often glycated, composed of a single subunit, and associate into trimers [15]. The small 2 S albumin proteins belong to the prolamin superfamily, contain a conserved set of cysteine residues, and are commonly cleaved into two subunits [18,19]. These three conserved proteins (legumins, vicilins, and 2 S albumins) have been identified in both Gd and Gl protein preparations [4,5], although the pattern of their distribution is not the same [20].

There are only a very limited number of past reports implicating traditional Gd cottonseed as a potential source of allergens. For example, a food supplement containing cottonseed was shown to generate positive skin prick test reactions in seven subjects who had experienced allergic reactions after ingesting the supplement in the late 1980s, and two of these subjects had severe reactions in a double-blind, placebo-controlled food challenge with cottonseed flour [21]. Skin prick testing of a single patient with elevated serum IgE and a history of eosinophilic esophagitis using 44 food extracts revealed positive reactions to garlic and cottonseed extracts in a single case study [22]. Further, consumption of a whole grain bread containing cottonseed in an otherwise healthy individual led to a severe allergic reaction, and a subsequent evaluation of the patient indicated a high level of circulating IgE antibodies against cottonseed protein [23]. A few more recent studies have raised controversial questions about the possible allergen risk in Gl cottonseed [24,25,26], but no laboratory or clinical studies have been published. While Zhang and Wedegaertner [26] raised concerns about possible allergenic reactions, Kumar and his colleagues [25] argued that cottonseed is not reported to instigate any allergic or hypersensitive immune response. Recently, novel peanut butter-like food products have been formulated from Gl cottonseed kernels [27,28]. For this reason, there is an urgent need to provide convincing experimental data to address allergy concerns from new Gl cottonseed-based food products. Thus, to experimentally assess the allergen risk in Gl cottonseed and identify potential allergens, protein isolates were evaluated in this study to examine cross-reactive binding by IgE from peanut and tree nut allergic samples and to identify IgE cross-reacting Gl cottonseed proteins.

## 2. Results

### 2.1. Cottonseed Protein Extract Cross-Reacts with Peanut and Tree Nut IgE

**Figure 1:**
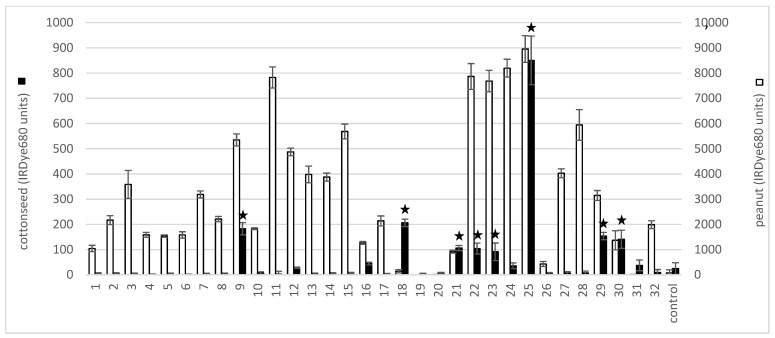
Glandless cottonseed protein cross-reacts with peanut and tree nut allergic volunteer IgE. ELISA binding to Gl cottonseed or peanut proteins was evaluated with peanut and tree nut allergic volunteer sera. IRDye680 units representing IgE binding to cottonseed protein (black bars) are indicated on the left side *Y*-axis and IgE binding to peanut protein (white bars) on the right side *y*-axis. Volunteer number is indicated on the *X*-axis. Data represents the average of at least four replications per sample with standard deviation shown as ± error bars. Bars with stars over them indicate values greater than two standard deviations above the mean.

IgE binding to glandless cottonseed protein was evaluated by ELISA with 32 peanut and/or tree nut allergic samples. Eight of the thirty-two samples tested produced an IgE signal that was greater than two standard deviations above a control nonallergic sample (Figure 1). Six of the eight positive samples had peanut ImmunoCAP scores (in CAP kU/L) of 2.9 or higher including sample 25, and for two samples (18 and 30) peanut IgE ImmunoCAP scores were not known (Table 1). Sample 18 had ImmunoCAP scores of 18.9 to hazelnut, 25.4 to walnut, and 3.14 to pistachio, while sample 30 had a 15.5 value for pecan (Table 1). In most instances, binding of the eight positive cottonseed protein volunteer samples was much lower than the corresponding binding to peanut extract (Figure 1).

### 2.2. 49 and 51 kDa Cottonseed Proteins Cross-React with Peanut and Tree Nut Allergic IgE

**Figure 2:**
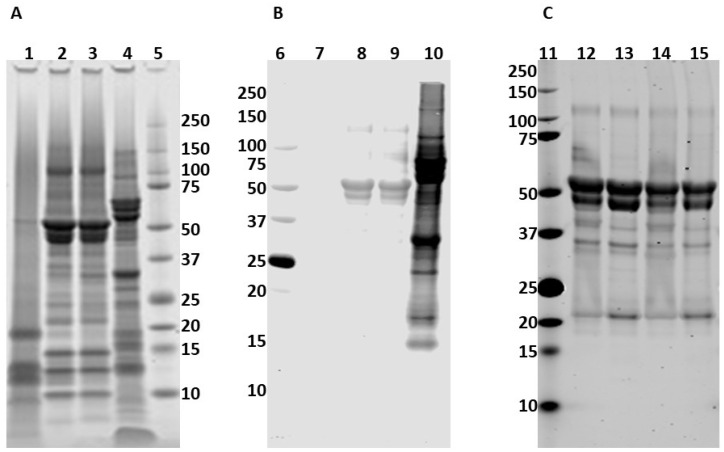
IgE from pooled peanut and tree nut sera recognize glandless cottonseed proteins. Three Gl cottonseed protein samples (Glw, Gla, & Gli) were compared to peanut extract by SDS-PAGE (**A**) and immunoblot with pooled sera from eight ELISA positive samples (**B**). IgE binding to Gla and Gli proteins was evaluated with and without DTT treatment (**C**). Glw protein is shown in lanes 1 and 7, Gla proteins in lanes 2, 8, 12, and 13, and Gli proteins in lanes 3, 9, 14, and 15, with peanut extract in lanes 4 and 10. DTT treated Gla and Gli treated proteins are in lanes 13 and 15. Molecular weight markers are shown in lanes 5, 6, and 11.

To determine which peanut and tree nut allergic IgE reactive proteins were within the cottonseed protein preparation, the eight volunteer samples that recognized Gl cottonseed extracts were pooled and used to probe a Western blot. Three different Gl cottonseed protein samples were used to compare IgE binding to whole peanut extract by immunoblot (Figure 2). Sequentially extracted water- and alkali-soluble fractions of cottonseed protein from defatted Gl cottonseed meal were tested along with alkaline extraction alone. SDS-PAGE revealed that the water extract (Glw) contained mostly lower molecular mass proteins, while both the sequential (Gla) and alkaline only (Gli) extracts contained several proteins migrating in the range of 10–150 kDa (Figure 2). While the Glw extract was not recognized by peanut and tree nut allergic IgE, both the sequential Gla and alkaline only Gli extracts bound IgE. However, the binding to the cottonseed extract was much less intense when compared to peanut extract binding on the same blot (Figure 2B) consistent with what was observed with the ELISA. IgE binding was further assessed using only the sequential and alkaline extracts on the same blot, and there were two primary bands within the Gla and Gli isolates migrating near the 50 kDa marker (at approximately 49 and 51 kDa) that were recognized by the pooled allergic samples (Figure 2). Following sample treatment with a reducing agent (dithiothreitol, DTT), IgE signal to a minor band migrating near 25 kDa was more pronounced, but the signal to the 51 and 49 kDa bands remained near the same intensity (Figure 2).

### 2.3. Cottonseed Vicilin and Legumin Proteins Cross-React with Peanut and Tree Nut IgE

**Figure 3:**
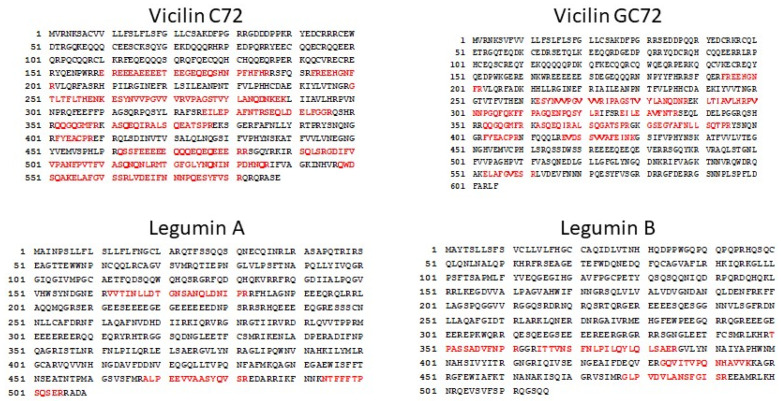
Peptides From the 49 and 51 kDa Bands Matching Cotton Vicilin and Legumin Proteins. Mass-spectrometry identified peptides from the 49 and 51 kDa bands that matched sequences from the cotton C72 and GC72-A vicilin and the legumin A and B proteins are colored red.

Bands corresponding to the 51 and 49 kDa IgE-reactive bands were excised from SDS-PAGE (Figure 2A), digested with trypsin, and analyzed by liquid chromatography coupled mass-spectrometry. The mass-spectrometric analysis revealed peptides matching four proteins, two vicilins, and two legumins from each of the bands. There were 21 unique peptides in the 51 kDa band and 20 unique peptides in the 49 kDa band that matched the Gossypium hirsutum vicilin C72 (Table 2 and Figure 3). Similarly, there were 11 unique peptides in the 51 kDa band and 9 unique peptides in the 49 kDa band that matched the GC72-A vicilin. There were four peptides matching Legumin B in the 51 kDa band and two peptides matching legumin A, while there were two peptides from each legumin A and B observed in the 49 kDa band.

### 2.4. Cottonseed Vicilin and Legumin Contain Sequences Similar to Peanut and Tree Nut IgE Epitopes

Vicilin and legumin proteins from peanuts and tree nuts are common allergens, and IgE often cross-reacts with these proteins from different nut sources. The sequences of the cotton vicilin and legumin proteins were compared to common peanut and tree nut allergens. A protein BLAST analysis of the C72 vicilin sequence indicated that it was most similar to the other cotton vicilin GC72 (72% identity) (Table 3). The Jug r 2 walnut vicilin (46% identity) and the Car i 2 pecan vicilin (44% identity) were more similar in sequence to C72 than the hazelnut Cor a 11 (39%), peanut Ara h 1 (36%), pistachio Pis v 3 (33%), or cashew Ana o 1 (32%) vicilin proteins. Sequence comparison of the cotton legumins produced markedly different results. A BLAST analysis of the cotton legumin B protein indicated that it was most similar to the pistachio Pis v 2 allergen (54% identity) and hazelnut Cor a 9 (47%), while the black walnut Jug n 4 and pecan Car i 4 legumin allergens were both 46% identical (Table 4). The cotton legumin A, cashew nut Ana o 2, and English walnut Jug r 4 were all 45% identical to the cotton Legumin B, while peanut Ara h 3 was only 36% identical.

The potential of cotton vicilin and legumin protein sequences to cross-react with orthologous allergens was evaluated using the Immune Epitope Database (IEDB) epitope prediction tool [29]. Using a 70% homology cut-off value, the cotton vicilin and legumin proteins were found to have some sequences similar to published nut allergens epitopes. For example, two peptides from the GC72-A vicilin were matched to the IgE epitopes from the Ara h 1 peanut allergen, two others to the Jug r 2 English walnut allergen, and one from the soy beta-conglycinin alpha subunit (Table 5). Similarly, sequences in the cotton C72 vicilin were found to be similar to four Ara h 1 IgE epitopes (Table 5). The two cotton legumins also contained sequences similar to published IgE epitopes. The cotton legumin A had three sequences similar to IgE epitopes from the cashew nut Ana o 2 legumin, and one sequence similar to soybean, walnut, hazelnut, and almond allergens (Table 5). Cotton legumin B harbored five sequences similar to cashew Ana o 2 IgE epitopes, three to hazelnut Cor a 9, two to walnut Jug r 4, one to pistachio Pis v 5, and one to soybean Gly m 6 (Table 5).

### 2.5. Cottonseed Vicilin and Legumin Models Reveal Potentially Surface Exposed IgE Cross-Reactive Epitopes

**Figure 4:**
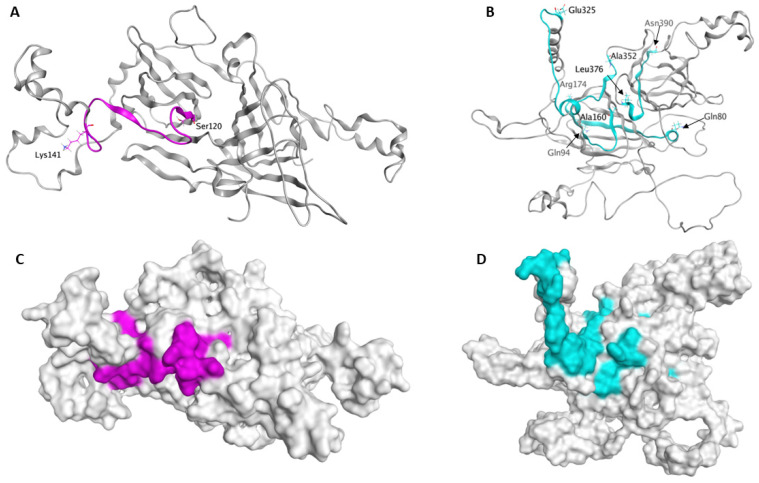
Cottonseed C72 Vicilin and Legumin B Models. Ribbon and space filling models of the cotton C72 vicilin (**A**,**C**) and legumin B (**B**,**D**) proteins. Peptides with at least 70% sequence similarity to peanut Ara h 1 epitopes are colored magenta in the C72 vicilin model while cross-reactive cashew nut Ana o 2 epitopes are colored cyan in the legumin B model.

The cotton vicilin A/B and legumin A/B proteins were likely recognized by IgE from peanut and tree nut allergic samples. Although there is not a clear pattern to the cross-reaction, the cotton vicilin A/B and legumin A/B proteins are similar to peanut and tree nut orthologs (Table 3 and Table 4). Further, analysis of the vicilin and legumin B proteins indicates sequence conservation among linear IgE epitopes mapped for some common peanut and tree nut allergens (Table 5). Models of the cotton vicilin and legumin proteins were generated to visualize the location of potentially IgE cross-reacting sequences. The cotton C72 vicilin was modeled using the Ara h 1 crystal structure as a template [30], and sequence with homology to an Ara h 1 epitope, ‘SMPVNTPGQFEDFFPASSRD’, was highlighted in the model (Figure 4). Similarly, the cotton legumin B protein was modeled based upon the Ara h 3 crystal structure [31]. Several sequences similar to epitopes from the cashew nut legumin Ana o 2, including the immunodominant epitopes ‘EESEDEKRRWGQRDN’ and ‘FQISREDARKIKFNN’, are highlighted in Figure 4. In each case, the model indicates that at least some part of each of the predicted epitopes is likely surface exposed.

## 3. Discussion

While eight foods (crustaceans, egg, fish, milk, peanuts, soy, tree nuts, and wheat) most commonly cause food allergy, numerous other foods have been identified as allergens [12,32]. Despite this, a majority of allergens arise from only a few protein families [33]. There are only a few reports of allergic responses to cottonseed, but among them a double-blind, placebo-controlled food challenge (DBPCFC) with cottonseed protein has provided strong evidence that cottonseed protein can be the causative agent for systemic reaction [21]. Although specific cottonseed proteins have not been identified as allergens, the IgE-binding and mass-spectrometry data, as well as the sequence analysis presented here provide a strong case that the cottonseed vicilin and legumin proteins may act as food allergens.

Cross-reaction among proteins occurs when an antibody recognizes both the original sensitizing allergen as well as sequences or structures from similar proteins. It has been suggested that 70% sequence homology could be considered as a marker for a high degree of cross-reaction among allergens, while cross-reactivity with 50% or less sequence homology may be less likely [34]. In general, the cottonseed C72 and GC72 proteins were below 50% identity to several peanut and tree nut vicilin and legumin allergens. Despite this, the cottonseed vicilin and legumin proteins are highlighted as potentially cross-reacting with IgE from peanut and tree nut allergic volunteers. Further support for this observation comes from the finding that cotton vicilin and legumin sequences share 70% similarity to several peanut and tree nut allergen IgE epitopes.

Like other seeds and nuts, cottonseed contains numerous proteins [5]. While a recent in silico analysis suggest the potential for cottonseed derived protein to be allergenic is low [24], the data presented here indicate care should be taken utilizing cottonseed protein as human food. Several other conserved plant allergens can be found in cottonseed including 2 S albumins, vicilin-like antimicrobial peptides 2-1/2, phosphoglycerate kinase, and protein disulfide-isomerase [4,5,20,33,35,36,37]. In peanuts and tree nuts, the 2 S albumin proteins are potent allergens [18,19]. The cotton 2 S albumins would be expected to be water soluble and migrate near 15 kDa marker, and there are bands migrating at that mass Glw protein sample, but no IgE binding was observed at that size in this analysis. Allergic volunteer donors have a specific pattern of allergen recognition within a given food. While some of the peanut and tree nut allergic volunteer samples used here appear to recognize the cottonseed vicilin and legumin proteins, it is possible that IgE from other food allergic volunteers may recognize other cottonseed proteins. Perhaps continued investigation with more diverse allergic volunteer samples and the incorporation of additional allergen evaluation methods, such as those recommended elsewhere (e.g., Guideline for the conduct of food safety assessment of foods derived from recombinant-DNA plants, https://www.who.int/docs/default-source/food-safety/food-genetically-modified/cxg-046e.pdf?sfvrsn=b4792881, accessed on 13 January 2023), would provide additional support to the findings presented in this study.

For every kilogram of fiber a cotton plant produces, it generates 1.6 kg of cottonseed. The relatively new development of Gl cottonseed presents a valuable and previously untapped source of food protein. Estimates suggest Gl cottonseed could provide as much as 10 billion kilograms of additional protein that could feed an estimated 590 million people [38]. While the use of Gl cottonseed as a triple-purpose commodity (fiber, feed, and food) crop will greatly increase net income for both cotton producers and processors [26], the best means to convey potential allergy risks and information for healthy and safe dietary choices related to Gl cottonseed is the use of accurate labelling steps and informed medical advice to sensitive populations [3]. Continued in-depth molecular and structural analysis of cottonseed proteins and peptides coupled with clinical studies will guide the safe and practical incorporation of Gl cottonseed products into a consumer nutrient food source as well as healthy food supplements [39].

## 4. Materials and Methods

### 4.1. Materials

Defatted peanut flour was obtained from Golden Peanut Company (Alpharetta, GA, USA). Novex tricine gels (10–20%), Novex NuPAGE LDS 4 X Sample Buffer, SimplyBlue SafeStain, bovine serum albumin (BSA, fraction V), and clear flat-bottom 96-well MaxiSorp plates were purchased from ThermoFisher Scientific (Grand Island, NY, USA). Precision Plus Protein Dual Color molecular weight standards were from Bio-Rad (Hercules, CA, USA). Volunteer peanut and tree nut allergic serum/plasma samples were obtained from PlasmaLab International (Everett, WA, USA). IRDye680RD or IRDye800CW labeled streptavidin and rabbit or mouse secondary antibodies were from LI-COR Biosciences (Lincoln, NE, USA). Biotinylated mouse antihuman IgE was from SouthernBiotech (Birmingham, AL, USA). Lyophilized sequencing-grade modified trypsin (Promega Corporation, Madison, WI, USA) was used according to the manufacturer’s instructions.

### 4.2. Gl Cottonseed Protein Preparation

Defatted Gl cottonseed meal was prepared from the “NuMex 15 GLS” variety provided by Cotton, Inc. (Cary, NC, USA) [28]. Three cottonseed extracts were prepared from Gl seeds using the protocol of He et al., 2021 [4]. Briefly, Gl cottonseeds were sequentially extracted first with distilled water for the ‘Glw’ extracts and followed by extraction with 15 mM NaOH for the alkaline ‘Gla’ extracts. Alternatively, a third distinct extracts was prepared with one-step alkaline extraction using 15 mM NaOH to generate the ‘Gli’ extracts. Proteins extracted in the supernatants were precipitated with titration to pH 4.0, 7.0, or 5.0 by addition of 1 M HCl for the Glw, Gla, and Gli, respectively. After centrifugation, precipitated proteins were freeze dried, and stored at −20 °C. Defatted peanut extracts were prepared as described in Mattison et al., 2019 [40], with the exception that sodium borate buffer (100 mM H_3_BO_3_, 25 mM Na_2_B_4_O_7_, 75 mM NaCl, pH 8.6) was substituted for phosphate buffer. Following resuspension in an appropriate buffer, sample protein concentrations were measured by absorbance at 280 nm (Nanodrop, ThermoFisher Scientific, Grand Island, NY, USA) and samples were either used immediately or stored at −80 °C.

### 4.3. Enzyme-Linked Immunosorbent Assay (ELISA)

ELISA binding to peanut or cottonseed protein with peanut and tree nut allergic serum samples was performed using methods described by Mattison et al., 2014 [41]. Briefly, high binding 96-well plates were coated with 4 μg of Gl cotton protein preparation per well in 50 µL ELISA coating buffer (15 mM Na_2_CO_3_, 35 mM NaHCO_3_, pH 9.6) overnight at 4 °C. The protein solution was then removed by aspiration and wells were blocked with 1% (*w*/*v*) BSA in phosphate-buffered saline (pH 7.4, PBS) containing 0.1% Tween-20 (PBST) for one hour at room temperature. Plate wells were washed three times with 200 µL PBST, and then 50 µL of individual peanut and/or tree nut allergic sample (diluted 1:5 in PBST) was added. Following one hour incubation at 37 °C, the nut allergic sample was removed from plate wells by aspiration, and wells were washed three times with 200 µL PBST. IgE-cottonseed protein isolate interactions were assessed by (1) one-hour incubation at 37 °C with 50 μL of biotinylated antihuman IgE (1:1000, Southern Biotech, Birmingham, AL, USA), (2) three 200 µL PBST wash steps, and (3) 30 min incubation at 37 °C with 50 µL of IRDye 800-labeled streptavidin (1:5000, LI-COR Biosciences). After three final 200 µL PBST washes, IgE signal was measured with an Odyssey CLX imaging system (LI-COR Biosciences). There were at least four replicates used for each sample to calculate binding signal (reported as mean ± standard deviation included as error bars). Binding to cottonseed protein isolate was considered significant if the mean binding signal of an individual allergic volunteer sample was greater than two standard deviations above the binding of a nonallergic control sample.

### 4.4. Sodium Dodecyl Sulfate-Polyacrylamide Gel Electrophoresis (SDS-PAGE)

Cottonseed protein samples were analyzed with tricine-buffered SDS-PAGE. Normalized protein content (10 µg) was loaded into gel wells after the addition of Novex NuPAGE 4X LDS sample buffer and heating at 55 °C for 5 min. Samples were electrophoresed at 120 V for 90 min in an XCell SureLock Mini gel rig (ThermoFisher Scientific, Grand Island, NY, USA) on Novex 10–20% tricine protein gels. Protein bands were visualized using SimplyBlue SafeStain, and gel images were collected with an Odyssey Clx instrument (LI-COR Biosciences).

### 4.5. Immunoblot

Immunoblot to identify and characterize peanut and tree nut allergic human serum IgE-reactive cottonseed proteins was performed using methods described by Mattison et al., 2014 [41]. Briefly, SDS-PAGE resolved Gl cottonseed protein was transferred to PVDF membrane using an iBlot system (Invitrogen, Waltham, MA, USA). Membranes were blocked for 1 h at room temperature with 1% (*w*/*v*) BSA in phosphate-buffered saline (pH 7.4, PBS) containing 0.1% Tween-20 (PBST), and then incubated with a pool of cottonseed reactive serum samples (diluted 1:5) for 1 h at room temperature. Membranes were washed (3 × 5 min) with 5 mL of PBST, then incubated for 1 h at room temperature with biotinylated antihuman IgE (Southern Biotech, Birmingham, AL, USA) diluted 1:1000 in PBST and washed three times as described above. Finally, blots were incubated for 30 min at room temperature with IRDye-800-labeled streptavidin (1:5000 in PBST), washed (3 × 5 min) with 5 mL of PBST, and IRdye-800 signal was visualized by scanning with an Odyssey CLx 195 instrument (LI-COR, Lincoln, NE, USA).

### 4.6. Liquid Chromatography Tandem Mass-Spectrometry (LC-MS/MS)

Liquid chromatography tandem mass-spectrometry (LC-MS/MS) was used to identify peptides within proteins corresponding to IgE-bound bands excised from cottonseed protein isolate electrophoresed on SDS-PAGE as described in Mattison et al. 2014 [42]. In short, excised gel slices were chopped into small pieces, rinsed with 100 µL water, rinsed with 100 µL of 100 mM ammonium bicarbonate, and then dehydrated by the addition of 100 µL of 100 mM ammonium bicarbonate containing 50% acetonitrile followed by drying in a speed vacuum. Protein in dried gel pieces was reduced by the addition of 50 µL of 50 mM ammonium bicarbonate containing 10 mM dithiothreitol (DTT), alkylated with 50 mM iodoacetamide, and then digested with 0.1 µg of sequencing-grade modified trypsin (Promega, Madison, WI, USA) overnight at 37 °C. Peptides collected in the supernatant and two washes with 50 µL of 25 mM ammonium bicarbonate were combined and dried in a speed vacuum. Dried peptides were resuspended with 20 µL of 5% formic acid and analyzed using an Agilent 1200 LC system, an Agilent Chip Cube interface, and an Agilent 6520 Q-TOF tandem mass spectrometer (Agilent Technologies, Santa Clara, CA, USA). Mass spectra were analyzed using Mascot software (Matrix Science, Boston, MA, USA) to identify peptide sequences and determine percent of protein coverage. Peptide sequences were searched using the annotated SWISS-PROT protein sequence database within the Viridiplantae (40, 925 sequences) taxonomy [43]. Peptide and fragment mass tolerances were set to 20 and 50 ppm, respectively, for searches with cysteine carbamidomethylation as a fixed modification and oxidation of methionine included as a variable modification. The digestion enzyme was specified as trypsin and up to two missed cleavages were allowed.

### 4.7. Protein Sequence Analysis and Epitope Prediction

Peanut and tree nut allergen sequences were gathered from the International Union of Immunological Societies (IUIS) website (http://allergen.org/, accessed on 12 October 2022). The basic local alignment search tool (BLAST) server at the National Center for Biotechnology Information (NCBI, https://blast.ncbi.nlm.nih.gov/Blast.cgi, accessed on 12 October 2022) was used to compare cotton vicilin and legumin protein sequences with peanut and tree nut allergen sequences [44]. Default BLASTP parameters were used with the ‘align two or more sequences’ option checked. Similarity of the cotton vicilin and legumin proteins to peanut and tree nut allergen IgE epitopes was evaluated using a 70% homology cut-off value at the Immune Epitope Database (IEDB) epitope prediction tool (https://www.iedb.org/, accessed on 12 October 2022) [29].

### 4.8. Protein Modeling

Models for the cotton C72 vicilin (P09801) and legumin B proteins were created using Molecular Operating Environment (MOE 2020.0901, Chemical Computing Group, Montreal, QC, Canada) software and were generated with the best fit templates using the Protein Data Bank (PDB) homology search application within MOE. The final C72 vicilin model (E value 1.6 × 10^−^^20^ and EHMMER 1.0 × 10^−^^48^) used the 3SMH.D PDB template molecule from the Ara h 1 (P41B clone) core region [30] and contained cotton vicilin residues 1–418. The final cotton legumin B model (E value 2.4 × 10^−41^ and EHMMER 8.9 × 10^−51^) contained residues 39–510 and used the Ara h 3 (3C3V.A) crystal structure as the template molecule [31].

## Figures and Tables

**Table 1:** ImmunoCAP Values for Peanut and Tree Nut Allergic Samples.

Volunteer	Peanut	Almond	Hazelnut	Brazil Nut	Cashew	Pistachio	Walnut	Macadamia Nut	Pecan	Soy
1	108.43	ND	0.28	0.1	ND	ND	0.1	ND	ND	ND
2	100	ND	ND	ND	ND	ND	ND	ND	ND	ND
3	100	0.75	2.14	0.12	5.07	5.75	0.21	0.14	0.06	5.74
4	140.6	ND	ND	ND	ND	ND	ND	ND	ND	4.234
5	100	26.6	18.7	0.53	0.66	3.2	17.1	3.27	4.46	8.25
6	100	14.7	44	9.85	44.2	44.3	49.7	5.86	32.5	5.314
7	100	2.97	32.7	5.28	17.1	26.7	7.46	ND	ND	8.09
8	100	0.27	5.9	ND	10.8	4.63	0.54	ND	ND	5.11
9 *	100	ND	100	ND	ND	ND	ND	ND	ND	38
10	100	ND	1.34	0.16	0.38	0.92	0.35	ND	ND	ND
11	15.19	ND	ND	ND	ND	ND	ND	ND	ND	ND
12	43.2	ND	ND	ND	ND	ND	ND	ND	ND	ND
13	0.35	ND	ND	ND	ND	ND	ND	ND	ND	0.422
14	>100	ND	ND	ND	ND	ND	ND	ND	ND	ND
15	ND	ND	ND	ND	51.4	ND	ND	ND	65.7	ND
16	1.678	ND	0.406	ND	ND	ND	ND	ND	ND	ND
17	90.8	ND	ND	ND	ND	ND	ND	ND	ND	ND
18 *	ND	ND	18.9	ND	ND	3.14	25.4	ND	ND	ND
19	6.2	ND	ND	ND	ND	ND	ND	ND	ND	ND
20	1.3	ND	ND	ND	ND	ND	ND	ND	ND	ND
21 *	2.9	ND	ND	ND	ND	ND	ND	ND	ND	ND
22 *	67.4	ND	ND	ND	ND	ND	ND	ND	51.8	4.67
23 *	47.4	ND	ND	ND	ND	ND	ND	ND	ND	ND
24	74	ND	ND	ND	ND	ND	ND	ND	ND	ND
25 *	90.8	ND	ND	ND	ND	ND	ND	ND	ND	ND
26	86.8	ND	11.7	ND	ND	ND	4.74	ND	ND	5.8
27	51.5	ND	20	ND	ND	ND	ND	ND	ND	1.66
28	42.67	ND	ND	ND	11.3	ND	ND	ND	ND	9.49
29 *	99.3	10.4	27.2	9.32	82.6	ND	24	ND	ND	13.2
30 *	ND	ND	ND	ND	ND	ND	ND	ND	15.5	ND
31	ND	ND	ND	ND	ND	ND	38.6	ND	ND	ND
32	41	ND	ND	ND	10.6	ND	ND	ND	2.22	4.34

Values in CAP kU/L (* Indicates sample with cottonseed protein IgE-binding signal > two standard deviations above control in Figure 1).

**Table 2:** Proteins Matching Peptides from 51 and 49 kDa Bands.

**51 kDa Band**								
**Accession**	**Score**	**Mass**	**Matches**	**Match (sig)**	**Sequences**	**Seq (sig)**	**emPAI**	**Description**
VCLB_GOSHI	8731	70,598	320	320	21	21	3.8	Vicilin C72 OS = Gossypium hirsutum OX = 3635 PE = 2 SV = 1
VCLA_GOSHI	886	71,861	52	52	11	11	1.24	Vicilin GC72-A OS = Gossypium hirsutum OX = 3635 PE = 3 SV = 1
LEGB_GOSHI	275	59,072	11	11	4	4	0.39	Legumin B OS = Gossypium hirsutum OX = 3635 GN = LEGB PE = 2 SV = 1
LEGA_GOSHI	226	58,902	4	4	2	2	0.18	Legumin A OS = Gossypium hirsutum OX = 3635 GN = LEGA PE = 2 SV = 2
**49 kDa band**								
**Accession**	**Score**	**Mass**	**Matches**	**Match (sig)**	**Sequences**	**Seq (sig)**	**emPAI**	**Description**
VCLB_GOSHI	9443	70,598	339	339	20	20	3.49	Vicilin C72 OS = Gossypium hirsutum OX = 3635 PE = 2 SV = 1
VCLA_GOSHI	911	71,861	43	43	9	9	0.96	Vicilin GC72-A OS = Gossypium hirsutum OX = 3635 PE = 3 SV = 1
LEGA_GOSHI	193	58,902	4	4	2	2	0.18	Legumin A OS = Gossypium hirsutum OX = 3635 GN = LEGA PE = 2 SV = 2
LEGB_GOSHI	95	59,072	3	3	2	2	0.18	Legumin B OS = Gossypium hirsutum OX = 3635 GN = LEGB PE = 2 SV = 1

**Table 3:** C72 Vicilin Sequence Homology.

Name	Source	Accession	Query Cover	E Value	Percent Identity
GC72	Cotton	A0A1U8LQ34	99%	0	72.39
Jug r 2 2.0101	English walnut	Q9SEW4	78%	1.00 × 10^−127^	45.53
Car i 2.0101	Pecan	B3STU4	84%	2.00 × 10^−120^	44.47
Cor a 11.0101	Hazelnut	Q8S4P9	82%	2.00 × 10^−107^	39.39
Ara h 1 (P41B)	Peanut	P43238	72%	1.00 × 10^−77^	35.87
Pis v 3.0101	Pistachio	B4X640	84%	1.00 × 10^−88^	32.96
Ana o 1.0101	Cashew	Q8L5L5	84%	1.00 × 10^−86^	31.89

**Table 4:** Legumin B Sequence Homology.

Name	Source	Accession	Query Cover	E Value	Percent Identity
Pis v 2.0101	Pistachio	B7P073	89%	3.00 × 10^−168^	54.06
Cor a 9.0101	Hazelnut	Q8W1C2	98%	1.00 × 10^−144^	47.49
Jug n 4.0101	Black walnut	A0A1L6K371	98%	8.00 × 10^−150^	46.32
Car i 4.0101	Pecan	B5KVH4	98%	3.00 ×10^−146^	46.15
Jug r 4.0101	English walnut	Q2TPW5	98%	5.00 × 10^−139^	45.42
Legumin A	Cotton	XP_016701249.1	90%	5.00 × 10^−138^	45.05
Ana o 2.0101	Cashew	Q8GZP6	97%	8.00 × 10^−141^	45.04
Ara h 3.0101	Peanut	O82580	92%	6.00 × 10^−98^	35.85

**Table 5:** Cotton Vicilin and Legumin Peptide Sequences 70% Similar to Peanut and Tree Nut IgE Epitopes.

Epitope	Antigen	Organism
GC72 vicilin		
VNTPGQFEDFFPASS	Ara h 1	Arachis hypogaea (peanut)
YAEIKRGAMMVPHYNSKATV	Jug r 2	Juglans regia (English walnut)
ARLARGDIFVIPAGHPIAIT	Jug r 2	Juglans regia (English walnut)
QDIFVIPAGYPVVVN	Beta-conglycinin alpha subunit 2	Glycine max (soybean)
**C72 vicilin**		
SMPVNTPGQFEDFFP	Ara h 1	Arachis hypogaea (peanut)
VNTPGQFEDFFPASS	Ara h 1	Arachis hypogaea (peanut)
PVNTPGQFEDFFPASSRDQS	Ara h 1	Arachis hypogaea (peanut)
SMPVNTPGQFEDFFPASSRD	Ara h 1	Arachis hypogaea (peanut)
**Legumin A**		
NQLDQMPRRFYLAGN	Gly m 6	Glycine max (soybean)
GDIIAFPAGVAHWSY	Jug r 4	Juglans regia (English walnut)
GDIIALPAGVAHWCY	Cor a 9	Corylus avellana (European hazelnut)
FQISREDARKIKFNN	Ana o 2	Anacardium occidentale (cashew)
LDRTPRKFHLAGNPK	Ana o 2	Anacardium occidentale (cashew)
QDRHQKIRRFRRGDI	Ana o 2	Anacardium occidentale (cashew)
QNQLDQVPRRFYLAG	Pru du 6	Prunus dulcis (almond)
**Legumin B**		
FGMIFPGCPSTYQEP	Gly m 6	Glycine max (soybean)
AFQISREEARRLKYN	Cor a 9	Corylus avellana (European hazelnut)
GDIIALPAGVAHWCY	Cor a 9	Corylus avellana (European hazelnut)
IESWDPNNQQFQCAG	Jug r 4	Juglans regia (English walnut)
PHWNLNAHSVVYALR	Jug r 4	Juglans regia (English walnut)
YANQLDENPRHFYLA	Cor a 9	Corylus avellana (European hazelnut)
AIPAGVAHWCYNEGN	Ana o 2	Anacardium occidentale (cashew)
LKWLQLSVEKGVLYK	Ana o 2	Anacardium occidentale (cashew)
LSVCFLILFHGCLAS	Ana o 2	Anacardium occidentale (cashew)
RWGQRDNGIEETICTMRLKENINDP	Ana o 2	Anacardium occidentale (cashew)
QFRCAGVALVRHTIQ	Ana o 2	Anacardium occidentale (cashew)
ERGVLQNNALMVPHWNFNAS	Pis v 5	Pistacia vera (pistachio)

## Data Availability

The data presented in this study are available upon request.
